# Thermal and Humidity Stability of Mixed Spacer Cations 2D Perovskite Solar Cells

**DOI:** 10.1002/advs.202004510

**Published:** 2021-05-06

**Authors:** Huayang Yu, Yulin Xie, Jia Zhang, Jiashun Duan, Xu Chen, Yudong Liang, Kai Wang, Ling Xu

**Affiliations:** ^1^ Wuhan National Laboratory for Optoelectronics China‐EU Institute and Renewable Energy Huazhong University of Science and Technology Wuhan 430074 P. R. China; ^2^ School of Physics and Electronics Huanggang Normal University Huanggang 438000 P. R. China; ^3^ School of Science Beijing Jiaotong University Beijing 100044 P. R. China

**Keywords:** 2D perovskites, orientation, quantum wells distribution, solar cells, spacer cations, stability

## Abstract

In this article, two different types of spacer cations, 1,4‐butanediamonium (BDA^2+^) and 2‐phenylethylammonium (PEA^+^) are co‐used to prepare the perovskite precursor solutions with the formula of (BDA)_1‐_ *_a_*(PEA_2_)*_a_*MA_4_Pb_5_X_16_. By simply mixing the two spacer cations, the self‐assembled polycrystalline films of (BDA)_0.8_(PEA_2_)_0.2_MA_4_Pb_5_X_16_ are obtained, and BDA^2+^ is located in the crystal grains and PEA^+^ is distributed on the surface. The films display a small exciton binding energy, uniformly distributed quantum wells and improved carrier transport. Besides, utilizing mixed spacer cations also induces better crystallinity and vertical orientation of 2D perovskite (BDA)_0.8_(PEA_2_)_0.2_MA_4_Pb_5_X_16_ films. Thus, a power conversion efficiency (PCE) of 17.21% is achieved in the optimized perovskite solar cells with the device structure of ITO/PEDOT:PSS/Perovskite/PCBM/BCP/Ag. In addition, the complementary humidity and thermal stability are obtained, which are ascribed to the enhanced interlayer interaction by BDA^2+^ and improved moisture resistance by the hydrophobic group of PEA^+^. The encapsulated devices are retained over 95% or 75% of the initial efficiency after storing 500 h in ambient air under 40 ± 5% relative humidity or 100 h in nitrogen at 60 °C.

## Graphical abstract

In this work, the mixed spacer cations 2D perovskite (BDA)_0.8_(PEA_2_)_0.2_MA_4_Pb_5_X_16_ films are obtained by employing two typical spacer cations, 1,4‐butanediamonium (BDA^2+^) usually for Dion‐Jacobson phase and 2‐phenylethylammonium (PEA^+^) for Ruddlesden‐Popper phase. The device with (BDA)_0.8_(PEA_2_)_0.2_MA_4_Pb_5_X_16_ film achieves the power conversion efficiency of 17.21% with excellent thermal and humidity stability.

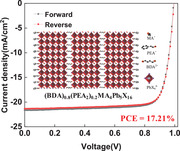

## 1 Introduction

Metal halide perovskites have received intense attention in the field of optoelectronics, such as photovoltaics,^[^ ^1^, ^2^, ^3^ ^]^ light‐emitting diodes,^[^ ^4^, ^5^ ^]^ and photodetectors,^[^ ^6^, ^7^ ^]^ attributed to the excellent optoelectronic properties, abundant raw materials, and low‐cost solution fabrication process.^[^ ^8^, ^9^ ^]^ Since the perovskites were first applied in photovoltaic devices in 2009,^[^ ^10^ ^]^ the power conversion efficiency (PCE) has been rapidly improved from less than 4% to over 25%.^[^ ^11^ ^]^ In the past few years, a lot of studies dedicated to resolving the problems of toxicity of lead,^[^ ^12^, ^13^ ^]^ solvents,^[^ ^14^ ^]^ and anti‐solvents^[^ ^15^ ^]^ have created valuable progresses, and then it is urgent to tackle the issue of perovskites stability. However, many factors including humidity, temperature, and ion migration can cause degradation of perovskites.^[^ ^16^ ^]^ Reducing the dimensionality of 3D metal halide perovskites to the 2D by introducing larger organic spacer cations of films has been confirmed as an effective method to enhance their stability. The presence of larger organic spacer cations in the films can inhibit ion migration and improve moisture and heat resistance.^[^ ^17^, ^18^, ^19^ ^]^

Before further discussions, the differences in structure and composition between 3D and 2D perovskites need to be clarified. For the 3D metal halide perovskites, the general formula is ABX_3_, where A is a monovalent cation, such as methylammonium (MA^+^), formamidinium (FA^+^), cesium (Cs^+^), or rubidium (Rb^+^), a divalent metal cation (Pb^2+^ or Sn^2+^), etc., as B and X for a halide anion (I^−^, Br^−^, or Cl^−^).^[^ ^20^ ^]^ In the case of 2D perovskites, there are three kinds of phase structure perovskites, they are Ruddlesden‐Popper (R‐P), Dion‐Jacobson (D‐J) and alternating cation in the interlayer space (ACI) phases, which are defined by the difference of spacer cations.^[^ ^21^ ^]^ Specifically, the R‐P phase perovskites with the formula S_2_A*_n_*_‐1_B*_n_*X_3_ *_n_*_+1_ (S means spacer cation) are obtained by adopting monovalent spacer cations like 2‐phenylethylammonium (PEA^+^)^[^ ^22^ ^]^ and *n*‐butylammonium (BA^+^),^[^ ^23^ ^]^ while D‐J phase counterpart with SA*_n_*_‐1_B*_n_*X_3_ *_n_*_+1_ is formed by divalent spacer cations, such as 1,4‐butanediamonium (BDA^2+^) or 3‐(aminomethyl) piperidinium (3AMP^2+^).^[^ ^24^, ^25^ ^]^ For the ACI phase perovskites, the only spacer cation is guanidinium (GA^+^) and the formula is (GA)(MA)*_n_*Pb*_n_*I_3_ *_n_*_+1_.^[^ ^26^ ^]^ For the R‐P phase perovskites, the hydrophobic groups of aromatic or alkyl amines strengthen the humidity stability. But the power conversion efficiency based on R‐P phase perovskites is limited by the factors including quantum wells with wider band gap, larger exciton binding energy (E_b_) as compared with 3D perovskites, and undesired in‐plane orientation.^[^ ^27^, ^28^ ^]^ The D‐J phase perovskites inherently exhibit better thermal stability attributed to the enhanced interlayer interaction.^[^ ^19^ ^]^ In addition, more uniform quantum wells and smaller E_b_ than the R‐P phase because of the shorter interlayer distance make it potential to achieve higher efficiency.^[^ ^29^ ^]^ Unfortunately, compared to the monovalent spacer cations like PEA^+^, the divalent spacer cations (such as BDA^2+^) have a risk of insufficient humidity stability.^[^ ^30^ ^]^ Usually, only one single type of spacer cation is employed in previous studies, while the mixed spacer cations specifically from different types has seldom been studied despite the huge potential.^[^ ^31^, ^32^ ^]^

In this work, two typical spacer cations, 1,4‐butanediamonium (BDA^2+^) usually for D‐J phase and 2‐phenylethylammonium (PEA^+^) for R‐P phase, were added to the precursor solutions, to synthesize the perovskite with the formula of (BDA)_1‐_ *_a_*(PEA_2_)*_a_*MA_4_Pb_5_X_16_ (*a* = 0, 0.1, 0.2, 0.3, 1; X = I^–^ or Cl^–^). The introduction of BDA^2+^ and PEA^+^ increased the degree of crystallinity and vertical orientation of the 2D perovskite films. As a result, a power conversion efficiency of 17.21% was achieved based on the device architecture of ITO/PEDOT:PSS/Perovskite/PCBM/BCP/Ag. Owing to the enhanced interlayer interaction by BDA^2+^ and the hydrophobic group of PEA^+^, the device showed both excellent thermal and humidity stability. Specifically, the encapsulated devices with (BDA)_0.8_(PEA_2_)_0.2_MA_4_Pb_5_X_16_ films retained over 95% or 75% of the initial PCE after storing 500 h in ambient air with humidity of 40 ± 5% or 100 h in nitrogen at 60 °C.

## 2 Results and Discussion

**Figure 1:**
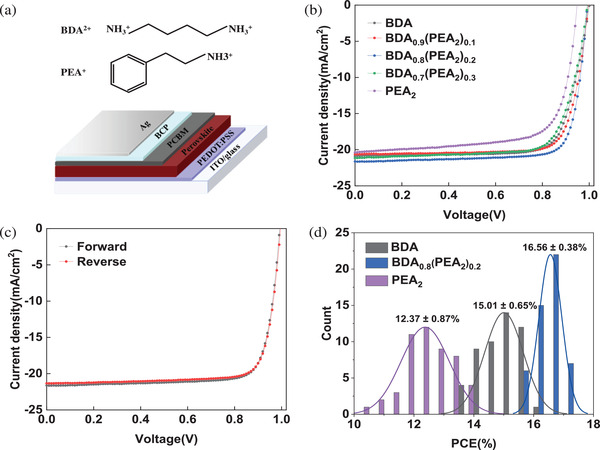
a) Chemical structure of PEA^+^ and BDA^2+^, device structure, b) *J–V* curves of devices, c) *J–V* curves of the best device with different scan directions, d) efficiency distribution histograms of 50 devices based on BDA, (BDA)_0.8_(PEA_2_)_0.2_, and PEA_2_ films, respectively.

Previous studies have proven the presence of contradiction in the efficiency and stability of 2D perovskite solar cells. Specifically, as the value of *n* (the thickness of the inorganic layer) increases, the band gap and E_b_ will be brought down to the level of 3D perovskites, which will lead to the increment of PCE but decrement of stability.^[^ ^33^ ^]^ The *n* value of the films is adjusted by the stoichiometric ratio in the precursor solution, where the commonly used choice is between 3 and 5.^[^ ^23^, ^34^ ^]^ As mentioned above, the choice of spacer cation types results in different 2D perovskites. The compositions of the D‐J phase and R‐P phase perovskites with *n* = 5 are defined as (BDA)MA_4_Pb_5_X_16_ and PEA_2_MA_4_Pb_5_X_16_ by introducing BDA^2+^ or PEA^+^ (structure shown in **Figure** **1**a). Thus, the ratio of two kinds of spacer cations is described by the formula (BDA)_1‐_ *_a_*(PEA_2_)_a_MA_4_Pb_5_X_16_, where *a* = 0 or 1 corresponds to pure D‐J or R‐P phase structure, and *a* = 0.1, 0.2, 0.3 corresponds to the mixed spacer cations. For the convenience of description, the composition of films is simplified to BDA, (BDA)_0.9_(PEA_2_)_0.1_, (BDA)_0.8_(PEA_2_)_0.2_, (BDA)_0.7_(PEA_2_)_0.3_, and PEA_2_ for *a* = 0, 0.1, 0.2, 0.3, 1, respectively. To evaluate the photovoltaic performance of perovskite films with different compositions, the photovoltaic devices were fabricated with the inverted planar architecture: Glass/ITO/PEDOT:PSS/Perovskite/PCBM/BCP/Ag. The current density versus voltage (*J*–*V*) characteristics of devices were measured under standard AM 1.5 G illuminated in Figure 1b. The photovoltaic parameters are summarized in **Table** **1**. In the case of *a* = 0 or 1, that is, D‐J or R‐P phase perovskite solar cells, the highest efficiency is 16.03% or 13.95%, respectively. Previously, a few of research reported that preferable orientation growth and large grain size of films was confirmed when a second spacer cation was introduced to 2D perovskite precursor solutions.^[^ ^31^ ^]^ Similar phenomena are observed in this work when the diamine BDA^2+^ and monoamine PEA^+^ cations were mixed to fabricate 2D perovskite films. For the scenario of mixed spacer cations, the device with (BDA)_0.8_(PEA_2_)_0.2_ film achieves the champion PCE of 17.21% with a short‐circuit current density (*J*_SC_) of 21.64 mA cm^–2^ and fill factor (FF) of 80.19%. The *J–V* curves of the device based on (BDA)_0.8_(PEA_2_)_0.2_ film with different scan directions show negligible hysteresis (Figure 1c). The details are shown in Table S1 (Supporting Information). Nevertheless, the excess PEA^+^ is harmful to the photovoltaic performance, evidenced by the PCE of the device with (BDA)_0.7_(PEA_2_)_0.3_ film is 15.70%. Besides, the repeatability was evaluated by testing 50 independent devices, with the results shown in Figure 1d. The external quantum efficiency (EQE) spectra and integrated current density can be seen in Figure S1 (Supporting Information).

**Table 1:** Photovoltaic parameters of the devices with different composition perovskite films

Composition		*V*_OC_ ^a)^ (V)	*J*_SC_ ^a)^ (mA cm^–2^)	FF^a)^ (%)	PCE^a)^ (%)
BDA	Best	1.00	20.9	76.67	16.03
	Average	0.99 ± 0.03	20.53 ± 0.75	75.66 ± 2.28	15.01 ± 0.65
(BDA)_0.9_(PEA_2_)_0.1_	Best	0.99	21.59	78.49	16.77
	Average	0.99 ± 0.01	21.18 ± 0.53	77.08 ± 2.08	16.12 ± 0.56
(BDA)_0.8_(PEA_2_)_0.2_	Best	0.99	21.64	80.19	17.21
	Average	0.99 ± 0.03	20.53 ± 0.75	75.66 ± 2.28	15.01 ± 0.65
(BDA)_0.7_(PEA_2_)_0.3_	Best	0.99	21.15	74.77	15.70
	Average	0.99 ± 0.03	20.53 ± 0.75	75.66 ± 2.28	15.01 ± 0.65
PEA_2_	Best	0.95	20.38	72.18	13.95
	Average	0.99 ± 0.03	20.53 ± 0.75	75.66 ± 2.28	15.01 ± 0.65

^a)^ The averages and standard deviations are calculated based on 50 devices.

**Figure 2:**
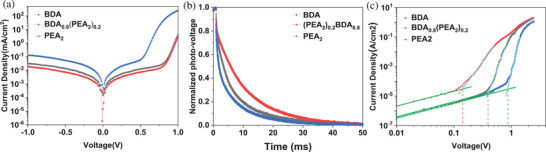
a) Dark current curves and b) transient photovoltage decay of devices, c) dark *J–V* curves of the hole‐only devices.

The measurements of dark current and transient photovoltage decay based on photovoltaic devices were performed to investigate the source of device performance differences. The device with (BDA)_0.8_(PEA_2_)_0.2_ film displays a smaller dark current (**Figure** **2**a), indicating the reduction of radiation loss.^[^ ^35^, ^36^ ^]^ The recombination time constants (*τ*_r_) were obtained by fitting the transient photovoltage decay curves (Figure 2b). The *τ*_r_ of (BDA)_0.8_(PEA_2_)_0.2_ device is 9.32 µs, while that of BDA (5.43 µs) and PEA_2_ (4.04 µs) device are shorter. The longer time constant is associated with less surface recombination, which contributes to higher efficiency.^[^ ^3^, ^37^, ^38^ ^]^ We derived trap density and charge carrier mobility from the dark *J–V* curves of the hole‐only devices (Glass/ITO/PEDOT:PSS/Perovskite/PTAA/MoO_3_/Ag) illuminated in Figure 2c. According to the space charge limited current (SCLC) method, trap density is expressed by Equation (1)

(1) $$N_{t}=\frac{2\varepsilon_{r}\varepsilon_{0}V_{\mathrm{TFL}}}{qL^{2}}\;$$

where *N*_t_ is trap density, *ε*_r_ is the relative dielectric constant, *ε*_0_ is the vacuum permittivity, *V*_TFL_ is trap‐filled limit voltage, *q* is the elemental charge and *L* is the thickness of films.^[^ ^8^, ^39^, ^40^ ^]^ The *ε*_r_ is calculated by Equation (2)

(2) $$\varepsilon_{r}=\frac{C_{g}L}{\varepsilon_{0}A}\;$$

where *C*_g_ is geometric capacitance, *L* is the thickness of the film, *ε*_0_ is the vacuum permittivity, *A* is the effective area of devices.^[^ ^41^ ^]^ In this test, the thickness of the perovskite layer (*L*) is about 350 nm, *A* is 0.055 cm^2^. The geometric capacitance is obtained from the high‐frequency range of the capacitance‐frequency curves (Figure S2, Supporting Information).^[^ ^42^ ^]^ The *C*_g_ of BDA, (BDA)_0.8_(PEA_2_)_0.2_, and PEA_2_ films are 2.45, 2.43, and 1.89 nF, and the calculated *ε*_r_ is 17.59, 17.46, and 13.56, respectively. The trap density of PEA_2_ film (1.05 × 10^16^ cm^–3^) is higher than that of (BDA)_0.8_(PEA_2_)_0.2_ (2.22 × 10^15^ cm^–3^) and BDA (6.32 × 10^15^ cm^–3^), which means that more non‐radiative recombination occurs at the interface or bulk of the film. The charge carrier mobility was calculated according to the Mott–Gurney Law (Equation (3)):^[^ ^8^, ^43^ ^]^

(3) $$J\;=\frac{9\varepsilon_{r}\varepsilon_{0}\mu V^{2}}{8L^{3}}\;$$

where *μ* is carrier mobility, *J* is current density and *V* is applied voltage. The hole mobility of (BDA)_0.8_(PEA_2_)_0.2_ film is 9.09 × 10^–3^ cm^2^ V^−1^ s^−1^, which is higher than that of BDA (8.06 × 10^–3^ cm^2^ V^−1^ s^−1^) and PEA_2_ (5.96 × 10^–3^ cm^2^ V^−1^ s^−1^), shown in Table S2 (Supporting Information). In the above‐mentioned device preparation process, the only variable is the chemical composition of the films, i.e., the type and ratio of spacer cations. Therefore, the difference in trap density and mobility depends on the crystalline quality, quantum wells distribution and orientation of the films, which will be discussed in detail below.

Herein, it is necessary to confirm whether the stoichiometric ratio of the components in the film is consistent with that in the precursor solution. The nuclear magnetic resonance (NMR) spectroscopy was employed to determine the composition of organic cations in the films (Figure S3, Supporting Information). The films were scraped off the substrate and dissolved in deuterated dimethyl sulfoxide. The types and ratio of organic cations can be obtained by integrating the corresponding peaks. The calculated ratio of organic cations is very close to that in the precursor solution.

Commonly, the R‐P phase films contain different *n* value 2D perovskites. Referable to the small‐*n* 2D perovskites with larger exciton binding energy and wider band gap, the corresponding exciton absorption peaks may be discovered in the absorption spectrum.^[^ ^44^ ^]^ Another common feature of R‐P phase film is the gradient distribution of the *n* value in the vertical direction, which means the *n* value of 2D perovskite grains in the films gradually increases from the bottom to the top.^[^ ^45^, ^46^ ^]^

**Figure 3:**
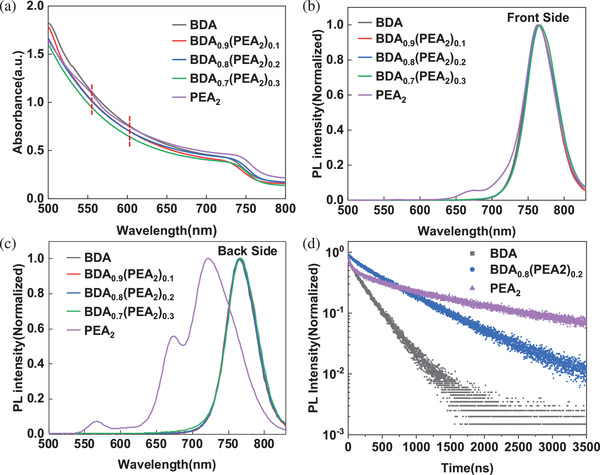
Optical spectroscopy of perovskite films. a) Absorption spectra, steady‐state PL spectra excited from b) front side and c) back sides, d) time‐resolved PL decays.

**Figure 4:**
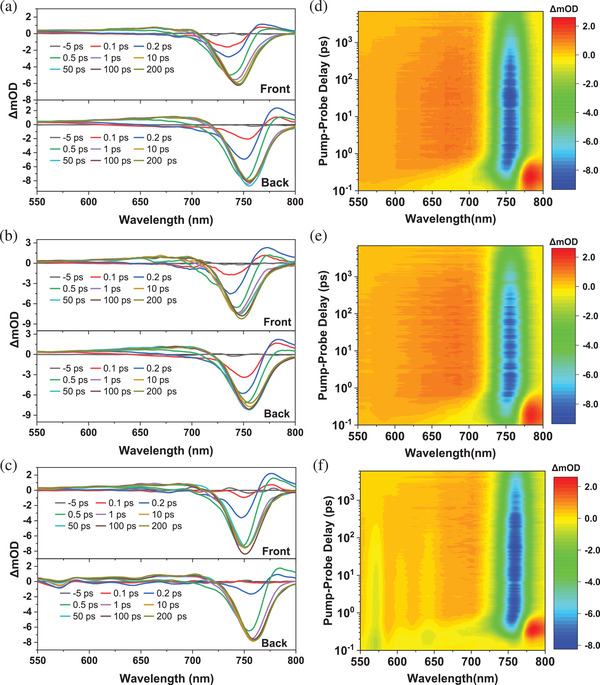
Transient absorption (TA) spectra perovskite films. Excited from the front side and back side for a) BDA, b) (BDA)_0.8_(PEA_2_), and c) PEA_2_ films at various delay times. Pseudocolor plots of TA excited from back side for d) BDA, e) (BDA)_0.8_(PEA_2_)_0.2_, and f) PEA_2_ films.

**Figure** **3**a shows the absorption spectra of perovskite films, only PEA_2_ film exhibits exciton peaks at about 550 nm (*n* = 2) and 600 nm (*n* = 3) as expected, while the BDA film does not. Despite the addition of PEA^+^, the exciton peaks of small‐*n* quantum wells do not appear in the mixed spacer cationic films, which indicates that their properties are similar to the BDA 2D film properties. This difference is more significant in the photoluminescence (PL) spectra excited from front and back sides of films in Figure 3b,c. For PEA_2_ film, significant blue shifting and extra emission peaks for small‐*n* 2D perovskites are observed when excited from the back. In contrast, the curves of BDA, (BDA)_0.9_(PEA_2_)_0.1_, and (BDA)_0.8_(PEA_2_)_0.2_ films excited from both sides are nearly coincident, which is consistent with the case of high phase purity or uniform distribution of different *n* value quantum wells.^[^ ^19^, ^22^ ^]^ In addition, just for (BDA)_0.7_(PEA_2_)_0.3_ films, there is a slight blue shifting in the range of 650–750 nm when excited from the back, which implies the formation of a small amount of small‐*n* R‐P phase perovskites. On the other hand, we compared the PL spectra of BDA and PEA_2_ films characteristics with *n* = 1, 3, and 5 (Figure S4, Supporting Information). The emission spectra of all BDA films are single emission peaks, and the curves excited from the front and back sides is basically the same. However, except for the case of pure phase with *n* = 1, PEA_2_ films show typical gradient distribution when *n* = 3 and 5. This proves that the BDA films tend to form uniform and concentrated quantum wells, rather than the gradient distribution like the PEA_2_ films. Therefore, when the concentration of PEA^+^ is high enough (such as (BDA)_0.7_(PEA_2_)_0.3_), small‐*n* R‐P phase perovskites will be formed at the bottom of the film, resulting in a blue shift in the PL Figure 3c. Time‐resolved photoluminescence (TRPL) measurements were performed to analyze the recombination dynamics of perovskite films (Figure 3d). The lifetimes of carriers were obtained by fitting the TRPL curves with a bi‐exponential decay function: *Y* = *A*_1_exp(−*t*/*τ*_1_)+*A*_2_exp(−*t*/*τ*_2_),^[^ ^47^, ^48^ ^]^ shown in Table S3 (Supporting Information). The short lifetime is attributed to defect‐assisted non‐radiative recombination that occurs on the surface of polycrystalline films, while the long lifetime corresponds to the recombination processes in bulk crystals with fewer defects.^[^ ^49^ ^]^ The *τ*_1_ (82.77 ns) and *τ*_2_ (693.23 ns) of (BDA)_0.8_(PEA_2_)_0.2_ film are significantly higher than the lifetime of BDA films with *τ*_1_ (33.45 ns) and *τ*_2_ (269.74 ns). This illustrates that the density of defect states decreased, meanwhile, the crystal quality is increased in the mixed spacer cationic films. It is worth mentioning that PEA_2_ films exhibit an overlong *τ*_2_ (1156.75 ns) but shows poor photovoltaic performance. Besides, the amplitude *A_1_* for the short lifetime *τ*_1_ (63.19 ns) is higher, which implies increased non‐radiative recombination. The surface morphology of the films obtained by atomic force microscopy (AFM) and scanning electron microscope (SEM) is shown in Figures S5 and S6 (Supporting Information). There are many pinholes or cracks in BDA and PEA_2_ films, while (BDA)_0.8_(PEA_2_)_0.2_ film is more uniform and dense. In Figures S5c,f and S6c,f (Supporting Information), it can be seen that there are micron‐sized crystals on the surface of the PEA_2_ film. This explains why the PEA_2_ film possesses a long *τ*_2_.^[^ ^48^ ^]^ However, according to the cross‐sectional SEM (Figure S6i, Supporting Information), these crystals do not penetrate the entire film, limiting the efficiency of charge transfer. We further investigated the carrier dynamics by the transient absorption (TA) measurements as shown in **Figure** **4**. For the BDA and (BDA)_0.8_(PEA_2_)_0.2_ films, only one bleach peak around 740–760 nm is detected whether excited from the front or back side. Three weak bleach peaks attributed to 2D perovskites with *n* = 2, 3, 4 at 570, 604, and 640 nm are observed when excited from the bottom in the case of PEA_2_ film. This is consistent with the results of absorption and PL spectra. Before 0.5 ps, the peak of PEA_2_ film at around 750 nm is significantly weaker compared with BDA and (BDA)_0.8_(PEA_2_)_0.2_ films, whether it is excited from the front or back side. After 0.5 ps, the peaks at 604 and 650 nm changed from negative to positive, and the peak at 750 nm began to increase rapidly. This indicates that the carriers of the small‐*n* phase (*n* = 3, 4) are transported to the large‐*n* phase (*n* = ∞), which is consistent with previous studies.^[^ ^50^, ^51^ ^]^ However, when *n* is further reduced, the efficiency of this process drops significantly. The signal at 570 nm (*n* = 2) decreases very slowly and does not become positive until about 100 ps. This kind of slow charge transport process is not observed in the TA spectrum of BDA or (BDA)_0.8_(PEA_2_)_0.2_ films, the only peaks of them at around 750 nm increases faster than PEA_2_ film. Compared with the gradient distribution, the carrier transport in the uniformly distributed quantum wells is more efficient.^[^ ^22^, ^51^ ^]^ Therefore, the devices based on (BDA)_0.8_(PEA_2_)_0.2_ perform a higher FF and PCE.

**Figure 5:**
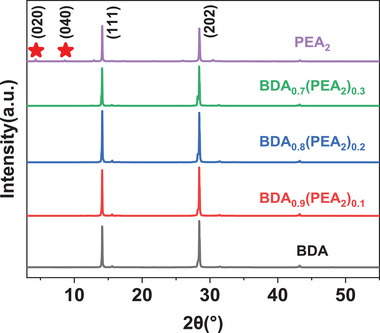
XRD patterns of (BDA)_1‐_ *_a_*(PEA_2_)*_a_*MA_4_Pb_5_X_16_ films.

Another property of 2D perovskite crystals is growth orientation. Due to the insulating nature of the organic spacer cations, the carriers are restricted in the quantum wells, hence the vertical growth orientation of the crystal is critical.^[^ ^23^, ^28^ ^]^ **Figure** **5** shows the X‐ray diffraction (XRD) patterns of (BDA)_1‐_ *_a_*(PEA_2_)*_a_*MA_4_Pb_5_X_16_ films. All the films exhibit two strong diffraction peaks at 14.2° and 28.4°, matching to (111) and (202) crystal planes, respectively. Among the samples, (BDA)_0.8_(PEA_2_)_0.2_ film performs the highest diffraction intensity, which means enhanced growth orientation toward the out‐of‐plane direction. However, PEA_2_ film displays two diffraction peaks at 4.2° and 8.6°, which is consistent with the (020) and (040) in‐planes orientation of 2D perovskites (*n* ≤ 2).^[^ ^3^, ^44^, ^52^, ^56^ ^]^ More detailed comparison is shown in Figure S5 (Supporting Information). The patterns of BDA, (BDA)_0.9_(PEA_2_)_0.1_, and (BDA)_0.8_(PEA_2_)_0.2_ films have three weak diffraction peaks at 5.5°, 8.5°, and 11.1°. These diffraction peaks, considered to correspond to perovskite in the D‐J phase, demonstrate that these films above have the same crystalline structure.^[^ ^29^, ^53^ ^]^ But, when the PEA^+^ ratio is higher, the (BDA)_0.7_(PEA_2_)_0.3_ film produces the same diffraction peak as the PEA_2_ film, caused by the formation of small‐*n* R‐P phase perovskites. This is consistent with the slight blue shift of (BDA)_0.7_(PEA_2_)_0.3_ PL curve in Figure 3. Consequently, (BDA)_0.8_(PEA_2_)_0.2_ is an ideal choice because it contains enough PEA^+^ while maintaining the D‐J phase crystal structure. In the case of PEA_2_ or (BDA)_0.7_(PEA_2_)_0.3_ films, excessive concentration of PEA^+^ will lead to small‐*n* 2D perovskites growing parallel to the substrate at the bottom of film, which explains the reason for poor PCE. The type and proportion of organic cations in the perovskite films may affect their energy level. According to the absorption spectra, the band gaps of the three films are obtained (Figure S8a). Combined with the test results of the Ultraviolet Photoelectron Spectrometer (UPS) (Figure S8b–d), the valence band, conduction band, and Fermi level of the film can be obtained (Figure S8e). Among them, the energy level of PEA_2_ film does not match the charge transport layers, which may be one of the reasons for its low *V*_OC_.

**Figure 6:**
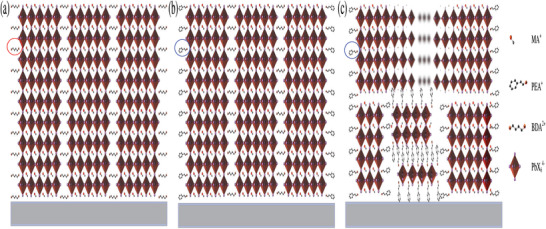
Schematic illustration of the perovskite film structure for a) BDA, b) (BDA)_0.8_(PEA_2_)_0.2_, and c) PEA_2_ films.

Based on the above information, schematic models of diverse composition films are established as illustrated in **Figure** **6**. For the R‐P phase 2D perovskite films, the crystallization process has been proven to starts from the gas–liquid interface.^[^ ^28^ ^]^ Attributed to the difference in formation energy, large‐*n* 2D perovskites will precipitate on the upper surface first.^[^ ^33^ ^]^ As a result, the increased spacer cations concentration at the bottom produce the formation of small‐*n* 2D perovskite, then the PEA_2_ film with gradient distribution quantum wells was obtained (Figure 6c). A similar situation occurs with long‐chain divalent spacer cations like 1,5‐pentamethylenediamine, 1,6‐hexamethylenediamine, but shorter ones such as BDA^2+^ and PDA^2+^ usually display uniformly distributed quantum wells,^[^ ^19^, ^24^ ^]^ as shown in Figure 6a. In the situation of (BDA)_0.8_(PEA_2_)_0.2_ film, we propose a self‐assembly crystallization process. At the initial stage, BDA‐based perovskite will first form nucleus and grow, because of the higher concentration. Then, with the consumption of BDA^2+^ and the evaporation of solvent, the concentration and the proportion of PEA^+^ will increase, thereby forming a PEA^+^‐rich shell on the surface of grains containing BDA^2+^. In this operation, the film crystallinity is increased and the surface defects of the crystalline grains are passivated effectively by PEA^+^, as shown in Figure 6b. But the proportion of the two spaced cations is very significant, because excess PEA^+^ will form a small‐*n* perovskite with undesired growth orientation at the bottom of the film.

**Figure 7:**
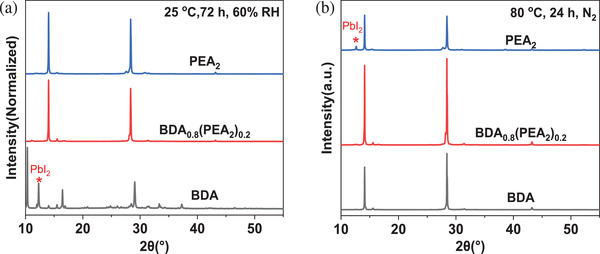
XRD patterns of BDA, (BDA)_0.8_(PEA_2_)_0.2_ and PEA_2_ perovskite films after stored a) in the ambient air at room temperature with ≈60% relative humidity for 72 h or b) in the glove box at 80 °C for 24 h.

The mixed spacer cation films possess thermal stability like D‐J phase perovskites and similar humidity stability to the R‐P phase. As shown in **Figure** **7**a, after stored in ambient air with a relative humidity of 60% for 72 h at room temperature, the BDA film was completely degraded, but (BDA)_0.8_(PEA_2_)_0.2_ film shows equivalent stability compared to the PEA_2_. Besides, the contact angles of water are 48.5°, 58.5°, and 69.3° for the BDA, (BDA)_0.8_(PEA_2_)_0.2_, and PEA_2_ films respectively, indicating the increased hydrophobicity with the introduction of PEA^+^ (Figure S9, Supporting Information). In Figure 7b, the PEA_2_ film (R‐P phase) decomposes and displays a diffraction peak of PbI_2_ at about 12.6° after continuous heating in nitrogen atmosphere at 80 °C for 24 h, while the BDA and (BDA)_0.8_(PEA_2_)_0.2_ films represent no signs of decomposition, suggesting better thermal stability.

**Figure 8:**
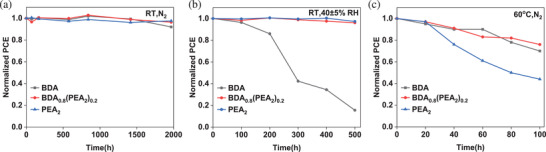
Stability test of photovoltaic devices with BDA, (BDA)_0.8_(PEA_2_)_0.2_, and PEA_2_ films in the dark. The devices were stored a) in the nitrogen glove box at room temperature, b) in ambient air with 40 ± 5% relative humidity at room temperature, or c) in the nitrogen glove box under 60 °C.

Furthermore, we performed the long‐term stability measurements of solar cell devices under different conditions (**Figure** **8**). The PCE of the devices based on different films did not decrease significantly after stored in a nitrogen glove box at room temperature for about 2000 h in dark. This indicates that the degradation caused by internal factors such as ion migration is negligible in above 2D perovskite solar cells.^[^ ^17^, ^54^, ^55^ ^]^ When stored in ambient air (relative humidity 40 ± 5%), the PCE of the device based on BDA film rapidly decayed to less than 20% of the initial value within 500 h. The device based on (BDA)_0.8_(PEA_2_)_0.2_ and PEA_2_ films displayed better environmental stability and the PCE of both remains above 95% of the initial. Besides, the devices were placed on a hot plate at 60 °C for thermal stability testing in nitrogen atmosphere. The PCE loss of device based on PEA is the most obvious, only ≈40% of the initial value is retained. The devices based on (BDA)_0.8_(PEA_2_)_0.2_ and BDA show similar thermal stability, and the PCE exceeding 75% and 70% of the initial are retained after 100 h, respectively. Therefore, humidity and thermal stability, as well as high efficiency, are obtained simply by mixing two different types of spacer cations, BDA^2+^ and PEA^+^.

## 3 Conclusion

In summary, we have demonstrated that the efficient 2D perovskite solar cells with more balanced stability can be obtained by mixing of the two different spacer cations, BDA^2+^ and PEA^+^, in the precursor solutions. On the one hand, the utilizing of mixed spacer cations increases the degree of crystallinity and enhances the vertical orientation of 2D perovskite films. The (BDA)_0.8_(PEA_2_)_0.2_MA_4_Pb_5_X_16_ film displays a small exciton binding energy, uniformly distributed quantum wells and more efficient carrier transport. On the other hand, BDA^2+^ is mainly concentrated in the crystal grains and PEA^+^ is distributed on the surface in the self‐assembled (BDA)_0.8_(PEA_2_)_0.2_ MA_4_Pb_5_X_16_ film. Therefore, both humidity and thermal stability are achieved as a result of the enhanced interlayer interaction by BDA^2+^ simultaneously, improving the moisture resistance by the hydrophobic group of PEA^+^. Consequently, we suggest that the simple strategy of mixing two different types of spacer cations, which achieves complementary advantages of D‐J and R‐P phase, is promising to further improve the performance and stability of 2D, even 3D perovskite optoelectronic devices.

## 4 Experimental Section

##### Materials

Lead iodide (PbI_2_), Methylamine Hydroiodide (MAI), Methylamine hydrochloride (MACl), 2‐Phenylethylamine hydroiodide (PEAI), and PEDOT:PSS 4083 were purchased from Xi'an Polymer Light Technology Corp. PCBM was purchased from Luminescence Technology Corp. MoO_3_, Isopropyl alcohol (IPA, ≥ 99.9%) and chlorobenzene (CB, 99.8%) were purchased from Sigma‐Aldrich. 1,4‐butanediamine (98%), hydroiodic acid (HI, ≥ 47.0%, with H_3_PO_2_ stabilizer), Methylamine solution (30–33% in ethanol), glacial acetic acid (99.5%) were purchased from Aladdin.

##### Synthesis of (BDA)I_2_

Hydroiodic acid and BDA with the molar ratio of 2:1 were mixed slowly in a round flask under stirring at 0 °C for 2 h. Then the mixed solution was concentrated using a rotary evaporator at 60 °C to obtain the powder of (BDA)I_2_. The product was washed three times with diethyl ether and dissolved in ethanol. After recrystallized three times, the powder of (BDA)I_2_ was dried under vacuum at 60 °C for 12 h.

##### Synthesis of MAAc

The synthesis method referred to prior reports.^[^ ^14^ ^]^ Glacial acetic acid was slowly added to a round‐bottomed flask containing excess methylamine solution (the stoichiometric ratio of acetic acid to methylamine was 1:1.5) at 0 °C. After 2 h of stirring in an ice bath, the mixed solution was evaporated at 80 °C for 1 h to obtain transparent viscous liquid (MAAc). The liquid product was frozen into solid state, then washed with diethyl ether and crystallized as the same as (BDA)I_2_. The product was dissolved in ethanol and evaporated at 80 °C for 1 h to obtain MAAc for the perovskite precursors solvent.

##### Precursor Solutions

The (BDA)MA_4_Pb_5_X_16_ and PEA_2_MA_4_Pb_5_X_16_ precursor solutions were prepared by mixing (BDA)I_2_, MACl, PbI_2_ (1:4:5 molar ratios) or PEAI, MACl, PbI_2_ (2:4:5 molar ratios) in MAAc with Pb^2+^ molar concentration of 0.45 m. The ratio of spacer cations of (BDA)_1‐_ *_a_*(PEA_2_)*_a_*MA_4_Pb_5_X_16_ (*a* = 0.1, 0.2, 0.3, 1) were controlled by mixing the above two solutions with volume ratios of 1‐*a*:*a*. The precursor solutions were stirred at 60 °C overnight.

##### Device Fabrication

ITO glass was cleaned by sequentially washing with detergent, deionized water, acetone and isopropanol (IPA). Before use, the ITO was cleaned with ultraviolet ozone for 30 min. PEDOT:PSS was spin‐coated on ITO substrates at 4000 rpm for 45 s followed by annealing on a hot plate at 120 °C for 30 min in ambient air. After that, the substrates and solutions were preheated at 100 °C for 5 min and 80 °C for 15 min in the glove box, respectively. Then the perovskite precursor solution was spin coated at 4000 rpm for 30 s and annealed at 100 °C for 10 min. The PCBM solution (20 mg mL^–1^ in CB) was then spin‐coated on the perovskite film at 2000 rpm for 45 s. Then the solution of BCP (0.5 mg mL^–1^ in IPA) was spin‐cast on top of the PCBM at 4000 rpm for 30 s. Finally, a 100 nm silver layer was thermally deposited as the cathode with an effective area of 0.055 cm^2^ under a vacuum of 5 × 10^–4^ Pa.

##### Measurements and Characterizations

The *J–V* characteristics were measured under illumination of AM 1.5 G (100 mW cm^−2^) by using a Keithley 2400 source meter. The steady‐state PL spectra and time‐resolved PL decays of the perovskite films were measured on an Edinburgh fluorescence spectrometer (FLS920) and HORIBA Scientific DeltaPro, respectively. EQE was measured on an EQE system (Enli Technology Co., Ltd.). Philips diffractometer (X'pert PRO MRD) was used to obtain the X‐ray diffraction data of perovskite films deposited on a ITO/PEDOT:PSS substrate. The morphology of perovskite films was characterized by AFM (Bruker, Dimension3100) and SEM (Carl Zeiss, GeminiSEM). The contact angles of perovskite films were measured by optical contact angle measuring and contour analysis systems (Dataphysics, OCA20). NMR was obtained by using NMR spectrometer (Bruker, Ascend TM 600 MHZ). TA spectra were performed by transient absorption spectrometer (Light Conversion, HARPIA‐TA). The thickness of the perovskite films was measured by a probe surface profiler (Bruker, DektakXT). Perovskite energy level was measured by UPS (Kratos, AXIS‐ULTRA DLD‐600W).

##### Statistical Analysis

In order to facilitate comparison, part of the data is normalized, such as the transient photovoltage decay in Figure 2b, the steady‐state PL in Figure 3b,c and Figure S4 (Supporting Information), TRPL, time‐resolved PL decays in Figure 3d, the XRD patterns in Figure 7a, and the PCE in Figure 8. In Figure 1d and Table 1, averages and standard deviations of photovoltaic parameters are calculated based on 50 devices. The results of TRPL curves fitting were obtained using OriginLab software. TA spectrum was extracted by CarpetView software. The NMR spectroscopy was obtained by MestReNova software. In addition, representative AFM and SEM images are shown in Figures S5 and S6 (Supporting Information), and the corresponding scale bars are placed in these pictures. The rest of the charts show the data directly obtained during the measurements.

## Conflict of Interest

The authors declare no conflict of interest.

## Supporting information

Supporting Information

## Data Availability

The data that support the findings of this study are available from the corresponding author upon reasonable request.
