# Thermal and Humidity Stability of Mixed Spacer Cations 2D Perovskite Solar Cells

**DOI:** 10.1002/advs.202201367

**Published:** 2022-04-14

**Authors:** Huayang Yu, Yulin Xie, Jia Zhang, Jiashun Duan, Xu Chen, Yudong Liang, Kai Wang, Ling Xu

*Adv. Sci*. **2021**, *8*, 2004510

DOI: 10.1002/advs.202004510

In the originally published version of this article, there are some errors in the data of Table 1. The correct Table 1 can be found below. The authors apologize for any inconvenience this may have caused.

**Table 1:** Photovoltaic parameters of the devices with different composition perovskite films

Composition		*V*_OC_ ^a)^ (V)	*J*_SC_ ^a)^ (mA cm^−2^)	FF ^a)^ (%)	PCE ^a)^ (%)
BDA	Best	1.00	20.9	76.67	16.03
	Average	0.99±0.03	20.53±0.75	75.66±2.28	15.01±0.65
BDA_0.9_(PEA_2_)_0.1_	Best	0.99	21.59	78.49	16.77
	Average	0.99±0.01	21.18±0.53	77.08±2.08	16.12±0.56
BDA_0.8_(PEA_2_)_0.2 _	Best	0.99	21.64	80.19	17.21
	Average	0.99±0.02	21.37±0.57	77.45±2.52	16.56±0.38
BDA_0.7_(PEA_2_)_0.3_	Best	0.99	21.15	74.77	15.70
	Average	0.95±0.03	20.96±1.04	71.34±4.80	14.46±1.31
PEA_2_	Best	0.95	20.38	72.18	13.95
	Average	0.89±0.05	19.88±1.01	69.10±2.62	12.37±0.87

^a)^ he averages and standard deviations are calculated based on 50 devices.

