# Supplementary material for: Thermal and Humidity Stability of Mixed Spacer Cations 2D Perovskite Solar Cells
Source: Adv Sci (Weinh). 2021 May 6;8(12):2004510. doi: 10.1002/advs.202004510 (PMC8224444; doi:10.1002/advs.202004510)
Supplement: Supplementary file 1 — Supporting Information [file ADVS-8-2004510-s001.pdf]

© 2021 Wiley-VCH GmbH

ADVANCED SCIENCE
Open Access

Supporting Information

for *Adv. Sci.*, DOI: 10.1002/advs.202004510

Thermal and Humidity Stability of Mixed Spacer Cations 2D Perovskite Solar Cells

*Huayang Yu, Yulin Xie, JiashunDuan, Xu Chen, Yudong Liang, Kai Wang, Ling Xu**

Copyright WILEY-VCH Verlag GmbH & Co. KGaA, 69469 Weinheim, Germany, 2020.

Supporting Information

Thermal and Humidity Stability of Mixed Spacer Cations 2D Perovskite Solar Cells

Huayang Yu [1,†], Yulin Xie[1,2,†], JiashunDuan[1], Xu Chen [1], Yudong Liang [1], Kai Wang[3], Ling Xu [1,]*

[1]*Wuhan National Laboratory for Optoelectronics, China-EU Insitute and Renewable Energy, Huazhong University of Science and Technology, Wuhan, 430074, P.R.China.*

[2]*School of Physics and Electronics, Huanggang Normal University, Huanggang 438000,P.R China*

[3]*School of Science, Beijing Jiaotong University, Beijing 100044, P.R.China*

[1]H Yu, J Duan, X Chen. Y Liang. Prof. L Xu
Wuhan National Laboratory for Optoelectronics,
China-EU Insitute and Renewable Energy,
Huazhong University of Science and Technology,
Wuhan, 430074, P.R.China
E-mail: xuling@mail.hust.edu.cn

[2]Dr. Y.Xie
School of Physics and Electronics,
Huanggang Normal University,
Huanggang 438000, China

[3]Prof. K Wang
School of Science,
Beijing Jiaotong University,
Beijing 100044, P.R.China

†Authors contributed equally

* Corresponding author: xuling@mail.hust.edu.cn;

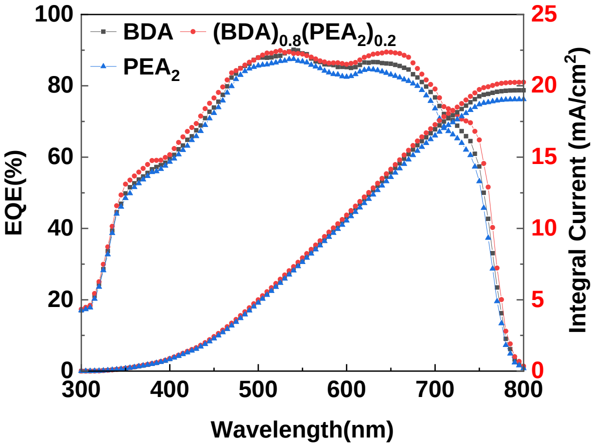

Figure S1. EQE and integrated current density of the devices based BDA,$(BDA)_{0.8}(PEA_2)_{0.2}$and PEA_2films.

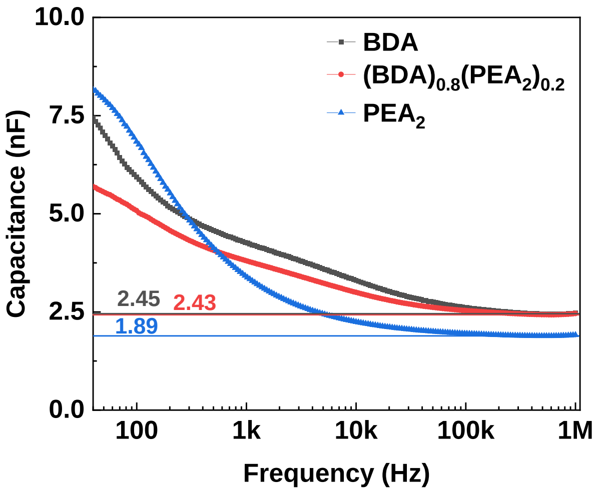

Figure S2.Capacitance-frequency curves of devicesbased BDA, $(BDA)_{0.8}(PEA_2)_{0.2}$and PEA_2films.

.

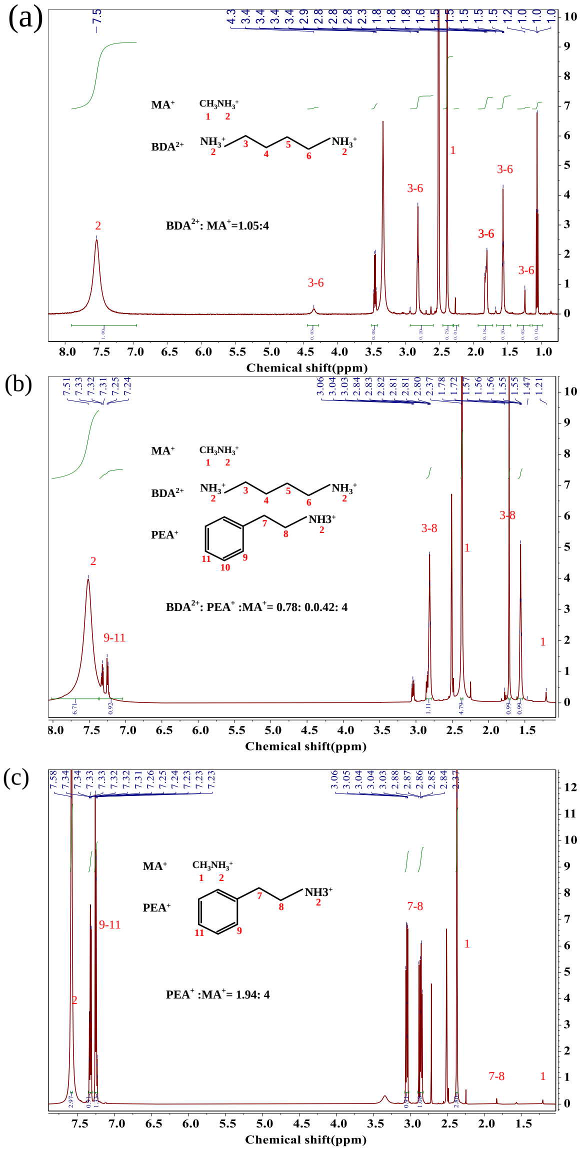

Figure S3. NMR spectroscopy of perovskite powder of $(BDA)MA_4Pb_5X_{16}$, $(BDA)_{0.8}(PEA_2)_{0.2}$ $MA_4Pb_5X_{16}$, and $PEA_2MA_4Pb_5X_{16}$.

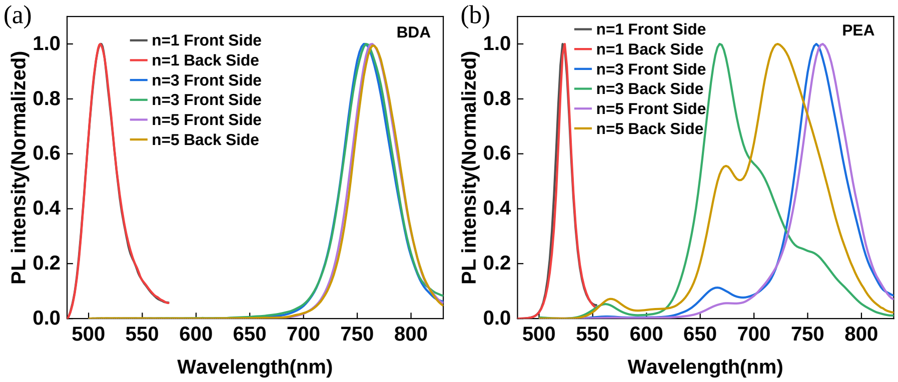

Figure S4.Steady-state PL spectra of perovskite films (*n* = 1,3,5) based on BDA or PEA_2 excited from front and back side.

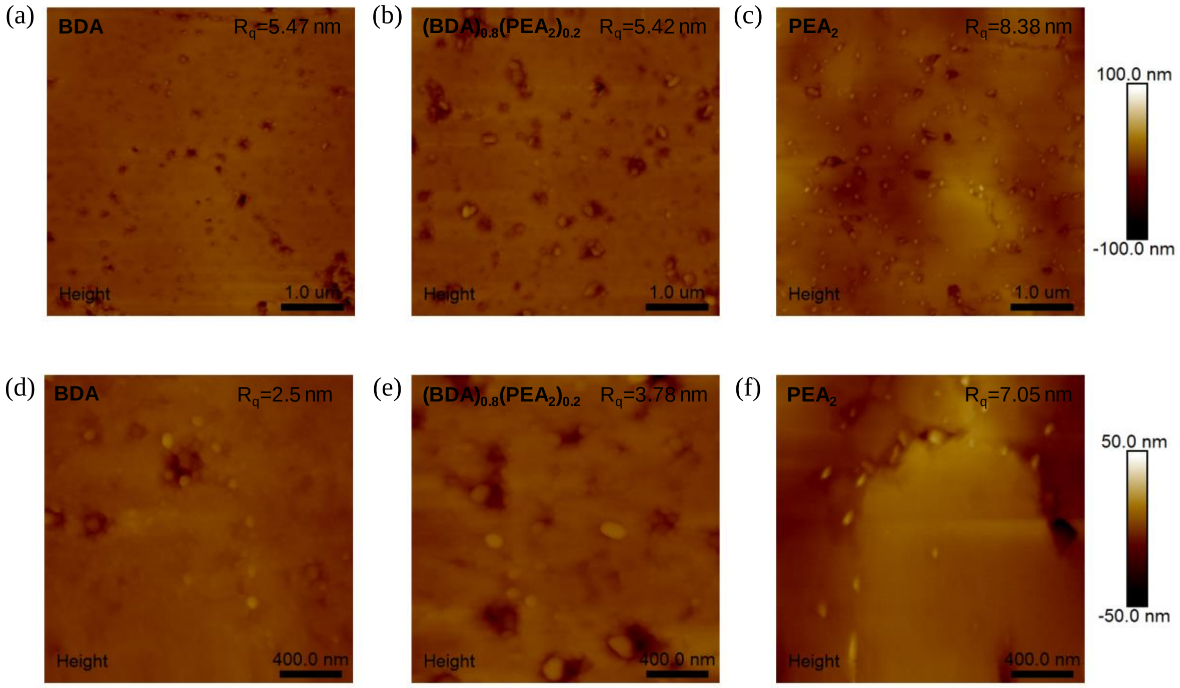

Figure S5. AFM images of BDA, $(BDA)_{0.8}(PEA_2)_{0.2}$ andPEA_2 films.

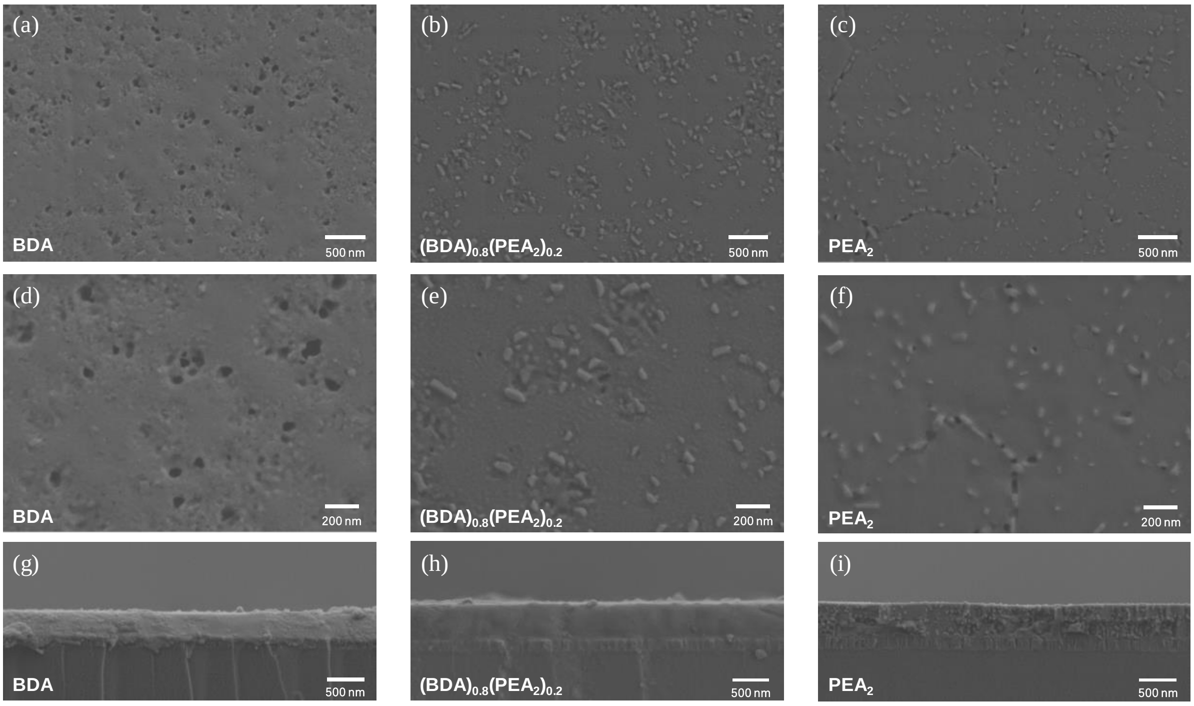

Figure S6. SEM images of BDA, $(BDA)_{0.8}(PEA_2)_{0.2}$ and PEA_2 films, (a)-(f) top-view SEM images, (g)-(i) cross-sectional SEM images.

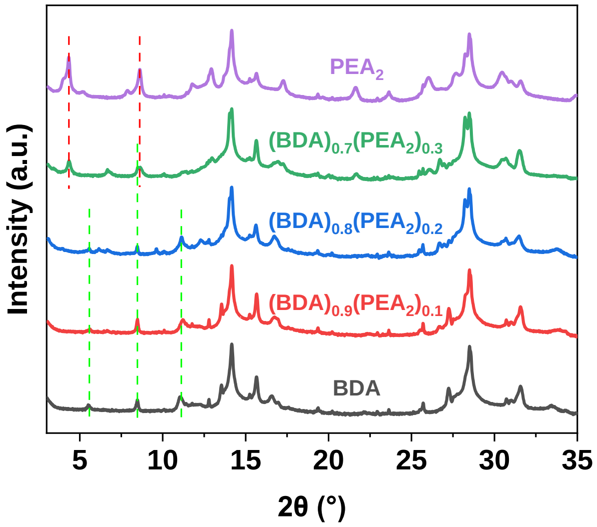

Figure S7. XRD patterns of $(BDA)_{1-a}(PEA_2)_aMA_4Pb_5X_{16}$films, the y-axis is logarithmic.

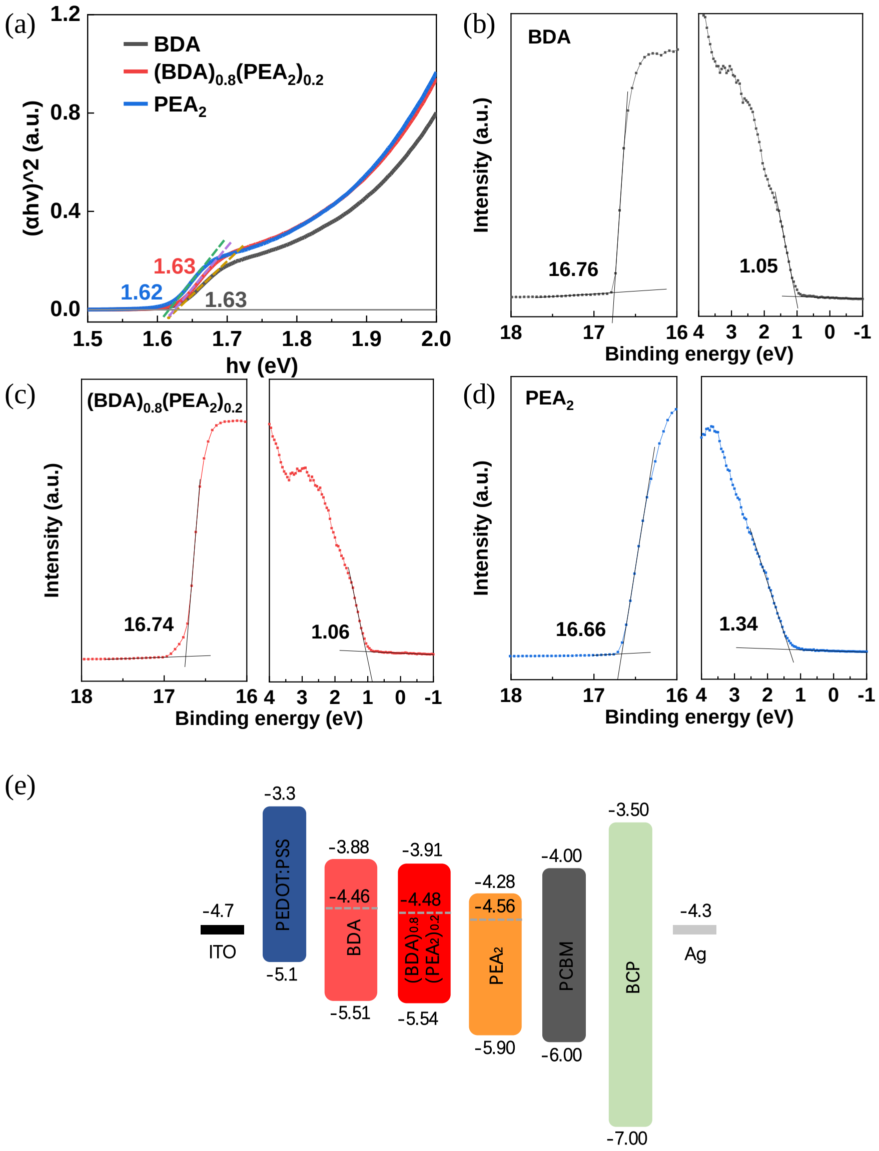

Figure S8.(a) Tauc plots of BDA, $(BDA)_{0.8}(PEA_2)_{0.2}$ and PEA_2 films, (b)-(d) UPS spectra of BDA, $(BDA)_{0.8}(PEA_2)_{0.2}$ and PEA_2 films, (e)Energy level diagrams of three types of perovskite films, charge transport layers and electrodes.

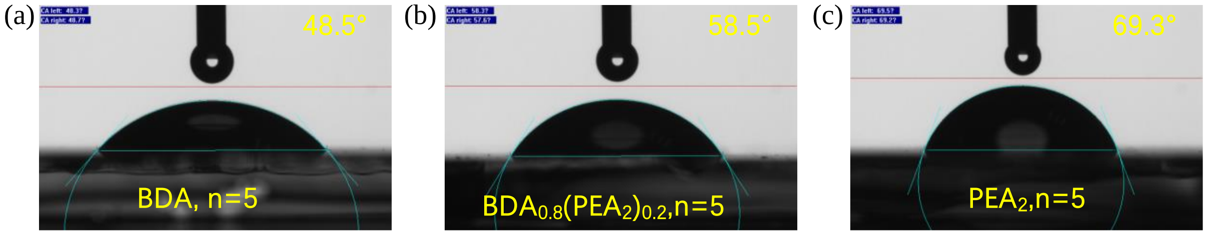

Figure S9.The contact angle of the perovskite films.

Table S1. Photovoltaic parameters of champion device with $(BDA)_{0.8}(PEA_2)_{0.2}PbX_3$ film.

Scan directions	V_{OC} (V)	J_{SC}(mA cm^{-2})	FF (%)	PCE (%)
Forward	0.99	21.64	80.19	17.21
Reverse	1.00	21.33	80.53	17.07

Table S2. Summary of trap density and charge mobility.

Composition	V_{TFL} (V)	N_t (cm^{-3})	μ (cm^2 V^{-1} s^{-1})
BDA	0.40	6.32×10^{15}	8.06×10^{-3}
$(BDA)_{0.8}(PEA_2)_{0.2}$	0.14	2.22×10^{15}	9.09×10^{-3}
PEA_2	0.86	1.05×10^{16}	5.96×10^{-3}

Table S3. Fitted lifetimes of carriers by TRPL measurements.

Composition	A_1	τ_1 (ns)	A_2	τ_2 (ns)	τ_{ave} (ns)
BDA	0.26	33.45	0.74	269.74	259.87
$(BDA)_{0.8}(PEA_2)_{0.2}$	0.16	82.77	0.84	693.23	679.66
PEA_2	0.61	63.19	0.39	1156.75	1070.67
